# 

**Published:** 1891-08

**Authors:** 


### CONTENTS—AUGUST, 1891.—VOL. VII., NO. 2.

ORIGINAL ARTICLES—

A Plea for the More General Use of Alkaloids. Vard H. Hulen, M, D., Galveston ..... 39

Some Practical Observations on the Management of Albuminuria in Pregnancy. J. F. Y. Paine, M. D., Galveston ..... 45

Pure Phosphorus in Diseases of the Nervous System. Bat Smith, M. D ..... 48

A Country Doctor's Experience with Antipyretics. R. S. Gregg, M. D., Manor, Texas ..... 52

Supra-Pubic Lithotomy, a Case. Will B. Davis, M. D., Puebla, Col ..... 54

SOCIETY NOTES—

Association American Medical Editors ..... 56

New County Medical Societies ..... 56

Plains Medical Society ..... 57

Mississippi Valley Medical Association ..... 58

EDITORIAL—

A New and Remarkable Phase in Medical Practice ..... 59

MEDICAL NEWS AND MISCELLANY ..... 61

BOOK NOTICES ..... 69

PUBLISHER'S NOTES ..... 72

